# Missing PPI prescriptions while overprescribing?

**DOI:** 10.1007/s00228-023-03564-7

**Published:** 2023-09-19

**Authors:** Ingrid Schubert, Veronika Lappe, Ursula Marschall, Daniel Grandt

**Affiliations:** 1 PMV Forschungsgruppe, Faculty of Medicine and University Hospital Cologne, University of Cologne, 50931 Cologne, Germany; 2 Head of Department Medicine/Health Care Research, BARMER, 42285 Wuppertal, Germany; 3 Klinik für Innere Medizin I, Klinikum Saarbrücken gGmbH, 66119 Saarbrücken, Germany

Dear Editor,

With great interest, we read the comprehensive systematic review by Shanika et al. [1] which highlights the high consumption of proton pump inhibitors (PPI) by about a quarter of the adults (23,4%) worldwide. In their conclusions, the authors stress the need for a rational use, better monitoring, and deprescribing where indications are lacking. We fully agree with these conclusions, but we would like to add the following aspect: the lack of PPI despite indication.

For Germany, high treatment prevalence has been reported [2], especially in patients with polypharmacy and multimorbidity. For the latter, Lappe et al. reported a treatment prevalence with PPI of 52% and a high mean amount of 442 defined daily doses (DDD) in 2019, indicating continuous treatment or high daily doses [3].

Despite high treatment prevalence with PPI, undersupply can be observed, especially when patients use, e.g., simultaneously NSAID and anticoagulants or glucocorticoids. The risk for gastrointestinal bleeding is increased compared to monotherapy with only one drug group [4]. To minimize the risk for gastric ulcer and stomach bleeding during treatment with these drugs, the prophylactic co-prescribing of PPI or other antiulcer drugs is recommended [5].

In order to investigate the extent of undersupply, we analyzed the data of one of the largest health insurance funds (BARMER) in Germany. The data was accessed via the scientific data warehouse. We focused on insured persons > 17 years of age without a diagnosis of malignant neoplasm (ICD-10 code C00-C97) and a simultaneous prescription of NSAID with (i) anticoagulants or antithrombotic agents (*n* = 82,756) and (ii) glucocorticoids (*n* = 41,319) over a period of at least 28 days in 2021. Drugs were classified according to the anatomical therapeutic chemical (ATC) classification.

According to our data, 40.0% of those with a simultaneous treatment of NSAID with anticoagulants/antithrombotic agents had no co-prescribing of a PPI, and only 0.1% received other drugs for acid-related disorders (H2-receptor antagonists, misoprostol). A comparable amount of 39.2% did not receive an indicated PPI while using NSAID and glucocorticoids for a longer period of time. Women seem to be more likely to receive a PPI [6]. Age has a stronger influence on the co-prescribing of PPI in patients with glucocorticoids compared to the other combination of drugs (Fig. 1).

**Fig. 1:**
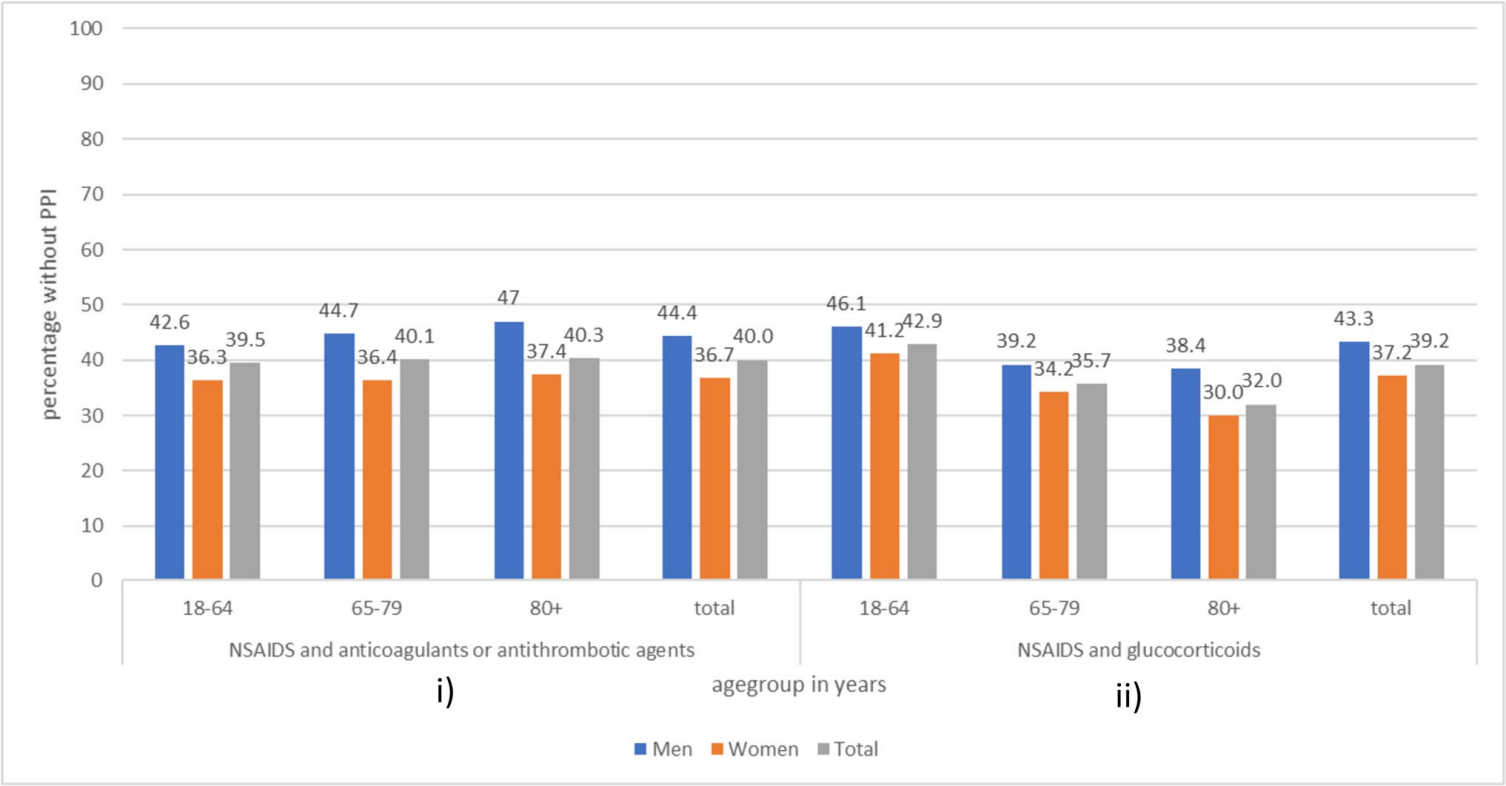
Percentage of the study population without PPI by insured persons with NSAID and simultaneous use of **i**) anticoagulants/antithrombotic agents or **ii**) glucocorticoids. **i**) Men (*n* = 35,395); women (*n* = 47,361); total (*n* = 82,756). **ii**) Men, 13,336; women, 27,983; total, 41,319. Data: scientific data warehouse BARMER; study population: insured persons > 17 years of age without a diagnosis of malignant neoplasm; simultaneous use over 28 days

In addition to the examples mentioned, there are further constellations where indicated PPI prescribing should be checked [5]. The strength of the study lies in the database covering about 12% of all statutory health-insured persons in Germany (9 million persons). Limitations are the lack of information on over-the-counter drugs and individual reasons for the use or nonuse of a drug.

The high treatment prevalence of PPI and long application time underline the need for a regular medication review as expressed by Shanika et al. [1]. International guidelines for the management of multimedication [7, 8] offer various instruments for medication assessment such as the Medication Appropriateness Index (MAI) [9]. While the risk of drugs and overuse in patients are frequently addressed, the possible underuse, i.e., a lack of a drug despite given indication, is less aware [10]. The German evidence-based guideline on multimedication [8] included underuse into the MAI as a key question when reviewing medication. As we could demonstrate for PPI, there may also be an undersupply of a commonly used medicine.

## Data Availability

Restrictions apply to the availability of these data. Data can be made available upon reasonable request and with permission of BARMER.
